# Flumazenil therapy for a gabapentin-induced coma: a case report

**DOI:** 10.1186/s13256-021-02816-3

**Published:** 2021-05-09

**Authors:** M. Masciullo, F. Pichiorri, G. Scivoletto, C. Foti, M. Molinari

**Affiliations:** 1 Spinal Cord Unit, IRCCS S. Lucia Foundation, Rome, Italy; 2 SPInal REhabilitation Lab (SPIRE), IRCCS Fondazione Santa Lucia, Rome, Italy; 3 Physical and Rehabilitation Medicine, University of Rome “Tor Vergata”, Rome, Italy

**Keywords:** Case report, Spinal cord injury, Spasticity, Neuropathic pain, Gabapentin, Baclofen, Flumazenil

## Abstract

**Background:**

Spasticity and neuropathic pain are common in patients after spinal cord injury and negatively affect patients’ quality of life. Gabapentin and baclofen are frequently used to treat these conditions. We present a flumazenil-reversed gabapentin-induced coma case, which, to our knowledge, is the second one described in scientific literature.

**Case presentation:**

A 70-year-old Caucasian man was admitted to our neurorehabilitation ward following a fall with cervical trauma that resulted in immediate tetraplegia. During his stay, he suffered from lower limb pain, both neuropathic and due to severe spasticity. Gradual baclofen and gabapentin administration was prescribed, with reduction in both pain and spasticity. One morning, the patient was found unresponsive, with a Glasgow Coma Score of 3. Head computerized tomography, electrocardiogram, electroencephalogram, vital signs, blood tests, breathing, and blood oxygenation were normal. Renal and liver failure were ruled out. Intravenous 0.25 mg of flumazenil (Anexate) was administered, resulting in complete neurocognitive recovery with a Glasgow Coma Score of 15.

**Discussion and conclusions:**

This case report highlights the importance of the individual response to certain pharmacological agents and suggests that further studies need to be conducted both on flumazenil and gabapentin pharmacodynamics to better understand their molecular–receptor activity, and on possible multiple flumazenil mechanisms of action, beyond its classical strict benzodiazepine antagonist action.

## Background

Following spinal cord injury (SCI), neuroplasticity, which involves neuronal, structural, and functional responses, is essential for recovery of neurological function, but the dark side of this neuroplasticity can be the development of neuropathic pain and spasticity. These common disabling conditions negatively affect mood, sleep, quality of life, and participation in activities, such as active recreation and employment. Neuropathic pain is present in 50–60% and spasticity in about 70% of individuals living with a SCI [1]. Gabapentin and baclofen are frequently used in the treatment of spasticity and neuropathic pain [2–6].

The present work describes a flumazenil-reversed gabapentin-induced coma case which, to our knowledge, is only the second one described in scientific literature, and suggests a possible expansion of flumazenil’s therapeutic indications beyond benzodiazepines.

## Case presentation

The case involves a 70-year-old Caucasian man who was admitted to our neurorehabilitation ward (Neurorehabilitation 1, Santa Lucia Foundation, Rome, Italy) following a fall with cervical trauma that resulted in immediate tetraplegia (AIS A, C5 level, according to the American Spinal Injury Association classification). Neurosurgical intervention was ineffective in decompressing the spinal cord, and no neurological recovery occurred. His medical history included stent-treated myocardial infarction, cholecystectomy, double biliary tract stenting, no smoking, no diabetes, sporadic and moderate alcohol intake prior to hospital admission, no renal or liver dysfunction, no epilepsy, and untreated spondylogenic myelopathy. Since cervical trauma, the patient had experienced neurogenic bladder and neurogenic bowel dysfunction. Chronic iron deficiency was observed in consequence to reduction in protein intake. The patient was a retired architect, and he was married, with no children. He did not have any substance abuse history.

On admission, the patient was alert, cooperative, and well oriented; he was classified as having tetraplegia (AIS A level C5) with marked spasticity in the lower limbs. Stage I sacral, stage III right heel, and stage II left heel bedsores were noted. Abdomen was plane, without any pain or discomfort with both superficial and deep palpation. Thorax was hypomobile with preserved vesicular murmur on pulmonary fields. Cardiac auscultation revealed a rhythmic heart rate, with free pauses and no abnormal heart sounds. The patient wore a condom catheter, which was removed to start the management of the neurogenic bladder through nurse-managed intermittent catheterization. Vital signs were blood pressure (BP) 113/65 mmHg, heart rate (HR) 52 beats per minute, oxygen saturation (SaO_2_) 98%, and temperature (T) 36 °C.

During his stay, he suffered from lower limb pain both neuropathic and due to severe spasticity, which is a frequent event in patients with spinal cord injury. A common and effective pharmacological approach for neuropathic pain and, in part, for spasticity, consists of gabapentin administration [2–5, 7]. Complete pharmacological therapy prior to the diagnosis was the following: intravenous sodium chloride 0.9% 500 ml twice per day, oral vancomycin 500 mg one-fourth of a vial four times per day, oral baclofen 25 mg three times per day (started on 21 December 2019), oral amiodarone (Cordarone) 200 mg per day, oral gabapentin (Neurontin) 300 mg twice per day, oral pantoprazole 40 mg per day, oral acetilsalicilic acid (Cardioaspirin) 100 mg per day, oral amlodipine 2.5 mg per day, oral potassium 600 mg twice per day, oral macrogol for bowel regularity, oral furosemide 25 mg per day, oral simethicone 80 mg three times per day, oral silodosin 8 mg per day, oral bisoprolol 25 mg per day, intravenous iron supplement 5 mg/5 ml one vial per day, subcutaneous fondaparinux 2.5 mg/0.5 ml per day, enemas to ensure regular defecation, oral probiotics, and oral zolpidem 10 mg only if needed to sleep (last intake more than 20 days before the onset of coma). Gabapentin was started on 15 January 2020 (orally 100 mg three times per day) and interrupted after 11 days because of drowsiness. Unfortunately, lower limb pain was so intense that gabapentin was once again prescribed on 24 February 2020, starting with a 300 mg dose in the evening. On 14 March 2020, dosage was increased to 300 mg twice a day (well tolerated by the patient), and gabapentin administration continued with this dosage until 22 June 2020.

On 22 June 2020, at approximately 7 am, the first round of drug administration took place and the patient received intravenous administration of sodium chloride (500 ml), an oral quarter of a 500 mg vial of vancomycin (for a *Clostridioides difficile* infection in resolution), oral 25 mg of baclofen, oral 300 mg of gabapentin, oral 40 mg of pantoprazole, oral 600 mg of potassium chloride, oral 25 mg of furosemide, oral 80 mg of simethicone, and intravenous 5 mg of iron supplement. The patient was alert and compliant and did not report any discomfort. After 1 hour, at 8 am, breakfast was distributed, and at that moment the nurse found the patient asleep and did not manage to wake him up through vocal recall nor by pain induction (nipple squeezing). Medical personnel were informed and immediately examined the patient. The patient was breathing normally, eyes closed. It was not possible to wake him up in any way. Vital signs were within normal range (blood pressure 125/73 mmHg, heart rate 50 bpm, SaO_2_ 97%, body temperature 36.5 °C), EEG showed normal alpha activity with basic rhythm at seven to eight cycles per second on the posterior and middle regions, symmetrical, medium voltage, stable. Urgent head CT did not show any sign of bleeding, urgent blood tests were normal without any sign of acute renal or liver failure or sign of infection (details are provided further on), and EKG was normal (apart from the known Q wave due to the previous myocardial infarction). The patient was clinically unresponsive with a Glasgow Coma Score (GCS) of 3. Intravenous hydration was administered, and a permanent urinary catheter was inserted to ensure renal function. At 11 am, after approximately 3 hours of coma, intravenous 0.20 mg flumazenil (Anexate) was administered. After 30 seconds, the patient opened his eyes and returned to his usual neurocognitive state, with a GCS of 15. As a precautionary measure, food supply was suspended for the following 6 hours. The patient was closely monitored for the rest of the day and did not present any other loss-of-consciousness episodes. Gabapentin dosage was reduced to 100 mg twice a day. The next day, the patient resumed his normal rehabilitation schedule.

Detailed results of blood tests performed on 22 June 2020:

White blood cells (WBC) 7.8 [n.r. 4.00–10.00] × 10^3^/mm^3^, hemoglobin (HGB) 9.10 [n.r. 13–17] g/dL, hematocrit (HCT) 28.30% [n.r. 40–49%], platelets (PLT) 185 [150–500] × 10^3^/mm^3^, mean corpuscular volume (MCV) 83.00 [n.r. 80–100] μm^3^, mean corpuscular hemoglobin (MCH) 26.80 [n.r. 27–32] pg, mean corpuscular hemoglobin concentration (MCHC) 32.10 [n.r. 32–36] g/dL, mean platelet volume (MPV) 10.50 [n.r. 6–11] μm^3^, procalcitonin (PCT) 0.19% [n.r. 0.15–0.50%], red cell distribution width (RDW) 17.60% [n.r. 11–16%], blood urea 28 [n.r. 16.6–48.5] mg/dL, creatinine 1.13 [n.r. 0.7–1.2] mg/dL , estimated glomerular filtration rate (eGFR) 65.4 [n.r. > 60] ml/min, glycemia 80 [n.r. 74–105] mg/dL, aspartate transaminase (AST) 20 [n.r. < 40] U/L, alanine aminotransferase (ALT) 20 [n.r. 1–41] U/L, gamma-glutamyl transferase (GGT) [n.r. < 60] 65 U/L, sodium 137 [n.r. 136–145] mmol/L, potassium 4.6 [n.r. 3.5–5.10] mmol/L, and C-reactive protein (CRP) 18.7 [n.r. < 5] mg/L; CRP was gradually returning within normal range after an increase due to *Clostridioides difficile* (on 15 June, CRP was 50.9 mg/L).

During the following 7 months, the patient stayed in our ward for rehabilitation and did not experience any other similar episode. He was discharged on 18 February 2021.

## Discussion and conclusions

This case report describes a flumazenil-reversed gabapentin-induced coma in an adult subject without specific organ dysfunction; to our knowledge, it is the second one described in scientific literature.

Gabapentin is an antiepileptic drug, widely used for the treatment of neuropathic pain and spasticity, which are frequent in patients with spinal cord injury [2–5]. It has a structure similar to that of GABA but does not bind to those receptors. The exact mechanism of the drug is relatively unknown, but it is thought to act at the alpha-2δ1 subunit of voltage-dependent calcium channels, thus inhibiting calcium currents [8]. Flumazenil (Ro15-178) is a 1,4-imidazobenzodiazepine with highly specific and competitive antagonist activity at benzodiazepine receptor [9]. It binds to the extracellular surface of GABA-A receptors and competitively displaces benzodiazepine molecules, preventing further benzodiazepine binding [10]. To our knowledge, only three gabapentin-induced coma cases have been reported: Buttler *et al*. [11], Dogukan *et al*. [12], Abdennour *et al*. [13]. The first two clinical cases were adults with known history of kidney failure, and the third one was a woman with diffuse subarachnoid hemorrhage complicated by seizures. As in the present case report, one of these patients was treated with flumazenil (in addition to dialysis) [11].

This case differs from the previous ones because this patient did not have renal failure (which could have caused a difficult urinary excretion of gabapentin), did not suffer from acute brain injury (as demonstrated by head CT), and did not suffer from cardiac arrhythmias (as demonstrated by EKG). Electrolyte imbalances were excluded by the blood panel. The patient was clinically in a coma state, but the EEG was normal. Therefore, the only plausible explanation to justify this sudden loss of consciousness could be a particular receptor sensitivity of the patient to the GABAergic activity of gabapentin or an accumulation effect or a mutual potentiation between baclofen and gabapentin. The clinical presentation was considered as a “benzodiazepine-like” overdose; therefore, an off-label intravenous injection of flumazenil was performed. Although flumazenil is considered a benzodiazepine-specific antidote, other literature case reports describe its use as an antidote for other drugs such as promethazine [14].

This case report highlights the importance of the individual response to certain pharmacological agents and suggests that further studies need to be conducted both on flumazenil and gabapentin pharmacodynamics to better understand their molecular–receptor activity, and on possible multiple flumazenil mechanisms of action, beyond its classical strict benzodiazepine antagonist action.

## Data Availability

Data available upon reasonable request.

## Abbreviations

SCI: Spinal cord injury
GCS: Glasgow Coma Score
CT: Computerized tomography
EKG: Electrocardiogram
EEG: Electroencephalogram
AIS: ASIA Impairment Scale
SaO_2_: Arterial oxygen saturation
GABA: Gamma-aminobutyric acid
P.O.: Per Os, oral administration
